# Patients', carers' and healthcare providers' views of patient‐held health records in Kerala, India: A qualitative exploratory study

**DOI:** 10.1111/hex.13721

**Published:** 2023-02-13

**Authors:** Linju Joseph, Sheila Greenfield, Semira Manaseki‐Holland, Lekha T. R., Sujakumari S., Jeemon Panniyammakal, Anna Lavis

**Affiliations:** ^1^ Institute of Applied Health Research, College of Medical and Dental Sciences University of Birmingham Edgbaston Birmingham UK; ^2^ Achutha Menon Centre for Health Science Studies Sree Chitra Tirunal Institute for Medical Sciences and Technology Trivandrum Kerala India

**Keywords:** healthcare communication, patient‐held records, patient safety, user perspectives

## Abstract

**Introduction:**

Poor medical information transfer across healthcare visits and providers poses a potential threat to patient safety. Patient‐held health records (PHRs) may be used to facilitate informational continuity, handover communication and patient self‐management. However, there are conflicting opinions on the effectiveness of PHRs, other than in maternal and child care. Moreover, the experiences of users of PHRs in low‐ and middle‐income countries are critical in policy decisions but have rarely been researched.

**Aim:**

This study aimed to explore similarities and differences in the perspectives of patients, carers and healthcare providers (HCPs) on the current PHRs for diabetes and hypertension in Kerala.

**Methods:**

A qualitative design was used comprising semistructured interviews with patients with diabetes/hypertension (*n* = 20), carers (*n* = 15) and HCPs (*n* = 17) in Kerala, India. Data were analysed using thematic analysis.

**Results:**

Themes generated regarding the experiences with PHRs from each user group were compared and contrasted. The themes that arose were organized under three headings: use of PHRs in everyday practice; the perceived value of PHR and where practice and value conflict. We found that in the use of PHRs in everyday practice, multiple PHRs posed challenges for patients carrying records and for HCPs locating relevant information. Most carers carried all patients' past PHRs, while patients made decisions on which PHR to take along based on the purpose of the healthcare visit. HCPs appreciated having PHRs but documented limited details in them. The perceived value of PHRs by each group for themselves was different. While HCPs placed value on PHRs for enabling better clinical decision‐making, preventing errors and patient safety, patients perceived them as transactional tools for diabetes and hypertension medications; carers highlighted their value during emergencies.

**Conclusion:**

Our findings suggest that users find a variety of values for PHRs. However, these perceived values are different for each user group, suggesting minimal functioning of PHRs for informational continuity, handover communication and self‐management.

**Patient and Public Involvement:**

Patients and carers were involved during the pilot testing of topic guides, consent and study information sheets. Patients and carers gave their feedback on the materials to ensure clarity and appropriateness within the context.

## 1 INTRODUCTION

Patient‐held health records (PHRs) contain patients' medical information documented by healthcare providers (HCPs) to reflect the healthcare services received by a patient.^1^ PHRs serve as a formal record for information sharing usually carried to healthcare visits by the patient or carers.^2^, ^3^ Usually, HCPs document health assessments, treatment plans and health services received by patients in these records.^4^ Despite low levels of evidence of improved maternal and child health outcomes,^5^ women, carers and HCPs value MCH records.^6^, ^7^, ^8^ However, there is limited evidence of improvement in health outcomes following the use of PHRs in noncommunicable diseases (NCDs) in low‐ and middle‐income countries (LMICs).^9^, ^10^

PHRs can be tailored for specific purposes such as to inform and involve patients in their care and to aid in self‐management, to improve handover communication and informational continuity of care across healthcare visits and HCPs.^2^, ^11^ The availability of patient medical information for HCPs forms the basis of informational continuity of care.^12^ With the increase of NCD burden in LMICs, patients requiring long‐term care and repeated encounters with HCPs are increasing.^13^ This in turn necessitates informational continuity for patient management and safety. A Mongolian study described the use of a paper‐based PHR (booklet) for information transfer across HCPs for patients with NCDs.^14^ PHRs also have significance for patients/carers with chronic NCDs as they need clear direction on optimal self‐management. There is limited literature regarding the usefulness of PHRs for patients with NCDs from LMICs.^15^

The National Programme for Prevention and Control of Cancer, Diabetes, Cardiovascular diseases & Stroke (NPCDCS) in India was introduced in recognition of the growing burden of NCDs. The programme has contributed to an improvement in screening facilities, access to and availability of medications for NCDs.^16^ However, there remain considerable gaps in both the patient awareness and patient control of major risk factors for NCDs such as diabetes mellitus and hypertension.^17^, ^18^ Kerala, the site of the current study, has been a relatively better‐performing state on health indicators such as mortality rates.^19^ This state has been experiencing a rapid epidemiologic transition, resulting in a huge burden of NCDs.^20^ Patients tend to visit different healthcare facilities (public and private) and HCPs, leading to fragmentation of medical information transfer across providers.^10^, ^21^ No paper‐based patient‐level facility‐based records are used in outpatient (OP) settings in public healthcare facilities in Kerala. A previous study in 2014 in Kerala found that disparate pieces of paper given to patients for patient‐held documentation were valued and kept safe by patients and when their use by clinicians was clear to patients, they carried them to all their clinic visits.^22^ Additionally, most patients in this study reported having little or no information about self‐management of their NCD.^22^ After reviewing the results, an experts' meeting suggested having a PHR for patients with NCDs to improve handover communication and informational continuity between providers and patients/carers in Kerala.^22^

Implementation of electronic health records in the public health system has become a priority in India.^23^ Within the context of the ongoing implementation of electronic health records in Kerala, it is important to investigate the use and perceived value of PHR for patients, carers and HCPs.^24^ The findings will guide policy decisions as to whether paper‐based PHRs should be continued or replaced by another system. Furthermore, given the prevalence of paper‐based PHRs in many LMICs, lessons learnt in one state in India would have implications throughout India and other LMICs.

Studies from HIC have reported on the patients', carers' and HCPs' perspectives on PHRs. A few qualitative studies have shown that patients valued having PHRs as a record of their condition. Other practical benefits of PHRs described by patients include the role as an aide‐memoire, as a tool for communicating with HCPs and in improving self‐management.^9^, ^11^ However, studies have also shown that when the intended purpose of the PHR is unclear to the users, then the value of PHRs is diminished for the users. Patients reported that when not having clarity on whose (patients'/HCPs') responsibility it is to record in PHRs or what the importance of information for patients/HCPs for self‐management or continuity of care is, PHRs are used suboptimally. HCPs' experiences in using PHRs from studies performed in HIC show that most HCPs appreciate the benefits of PHRs in improving the availability of medical information; however, the recording of PHRs and the use of PHRs by HCPs were low.^25^, ^26^, ^27^

On reviewing the literature, it is apparent that the experiences of the users of PHRs for chronic conditions from LMICs have received little attention. Improving patient and carer involvement in care is a potential approach to improve information transfer across different health settings and self‐management as well as seamless continuity of care. It is therefore important to understand how patients, carers and HCPs routinely use PHRs, or what value each group places on them if any. It is known that the benefits of a tool can arise due to the tool itself or due to its interaction with the wider context such as the conversations around it.^28^

Against this background, the qualitative study described here aimed to address a gap in the literature: the use and value of PHRs for patients', carers' and HCPs from LMIC. The study's purpose was to explore similarities and differences in the perspectives of patients, carers and HCPs on their perspectives of PHRs in Kerala. Three research questions were identified:

1. How do patients, carers and HCPs use current PHRs in Kerala, India?
2. What value do patients, carers and HCPs place on PHRs for themselves?
3. What are the users' perspectives on current PHRs' value for information transfer, handover communication and self‐management?

## 2 METHODS

### 2.1 Study design

This is an exploratory,^29^ descriptive^30^ qualitative study in which data were collected through semistructured interviews conducted with patients, carers and HCPs in OP settings in Kerala, India. We recruited patients with diabetes/hypertension as a tracer condition for NCDs.

### 2.2 Settings

We conducted the study in Kerala from February to November 2020. In 2017, the Government of Kerala initiated the ‘Aardram Mission’ to transform and mobilize the State's public healthcare system to meet the current health challenges. One of the objectives of the ‘Aardram Mission’ is the decentralization of healthcare from the secondary and tertiary levels to primary care‐led services and the initiation of population‐level activities to address the impact of NCDs, especially hypertension and diabetes.^31^, ^32^ Primary health centres (PHCs) have been upgraded to family health centres (FHCs) with additional HCPs.^13^, ^31^ FHCs have provision for treating patients with diabetes mellitus and hypertension under NPCDCS.^16^ Under this programme, free or subsidized medicines are available for NCDs such as diabetes, hypertension, cardiovascular and respiratory diseases in the public health facilities. Medicines for patients with diabetes and hypertension are dispensed monthly after consultation with the doctor at the PHC or FHCs. Currently, patient‐level electronic health records and health information systems are being installed in public healthcare facilities in Kerala.^31^ The electronic health records are longitudinal medical records that contain medical and demographic information about a patient, currently accessible to HCPs (public health facilities) only in Kerala.^33^ Given the plethora of types of private and public providers unconnected to each other, current electronic records planned in India for the public healthcare system do not solve the information exchange problem.

Both public and private sectors provide NCD services and can be described as follows: In the public health system, diabetes and hypertension services are provided at PHCs or FHCs. If specialist care is needed, patients are referred to NCD clinics at community health centres or district hospitals. In the private sector, general practitioners and health clinics (without in‐patient facilities) operate at the primary care level, and medium to large hospitals (both inpatient and OP facilities) operate at secondary and tertiary levels. Generally, public healthcare facilities are free of cost or charge minimally, while private healthcare facilities need patients/carers to pay for the healthcare services unless their insurance would pay.^21^ Few publicly financed health insurance schemes are designed to entitle poor and other vulnerable households to choose cashless healthcare from a pool of empanelled private or public providers. One such scheme for low‐salaried employees from the organized sector is the Employees State Insurance Scheme. Culturally, carers or family members are involved in a person's healthcare in India. Patients' spouses, children or other relatives accompany them when going for a healthcare check‐up or in the case of an emergency visit to a hospital. Carers discuss the healthcare condition of the patients with HCPs and HCPs hold discussions with patients and carers. In public health facilities, most patients or carers are given formal PHRs in which HCPs enter information to reflect the healthcare services received by a patient.^21^ These patient‐held documents take different forms such as outpatient (OP) tickets, diagnostic and lab reports, notebooks or patient passbooks or booklets (Table 1).

**Table 1:** Patient‐held health records available with patients in this study sample.

Patient‐held records used in outpatient settings in this sample of patients from Kerala	
Notebook	A plain notebook that is usually used for patients with chronic conditions such as diabetes mellitus (DM) or hypertension, mostly in the public health care facility, for HCPs to document their notes, medicines, etc.
NCD booklet or patient passbook from the family health centre	Printed NCD booklets distributed from the public health care settings. These booklets have additional information for patients such as the diet plan, recommended physical activity and health promotion messages to refrain from smoking.
Medical prescription and lab tests	Most HCPs in outpatient settings (both public and private) give patients a written or printed copy of their medication prescription. This usually contains the patient's demographic information, diagnosis and medication. Lab test results such as blood glucose results.
OP sheets/outpatient ticket or sheets	Patients visiting public health hospitals or health centres receive an OP ticket that is used by the HCP to write their notes (provisional diagnosis), medications and treatment plan for the patient.
Diabetes booklet from a specialized diabetes centre (private)	A patient‐held booklet with the doctor's notes, prescriptions and additional health care information for patients from a private specialty centre.

Abbreviations: HCP, healthcare providers; NCD, noncommunicable disease; OP, outpatient.

### 2.3 Study participants, sampling and recruitment

Three groups of participants were identified for the study: patients with diabetes and hypertension, carers and HCPs. In the pre‐COVID phase, the study was conducted at two FHCs in the Alappuzha district, Kerala, which is one of the first districts where the NPCDCS was implemented in 2015. The COVID‐19 lockdown and travel restrictions in Kerala, from March 2020, made onsite face‐to‐face interviews^34^ challenging. Therefore, from March 2020 to November 2020, telephone interviews were conducted with eligible participants from other districts (Trivandrum, Ernakulam, Malappuram and Wayanad) to capture views from a wider geographical area within Kerala.^35^

**Figure 1:**
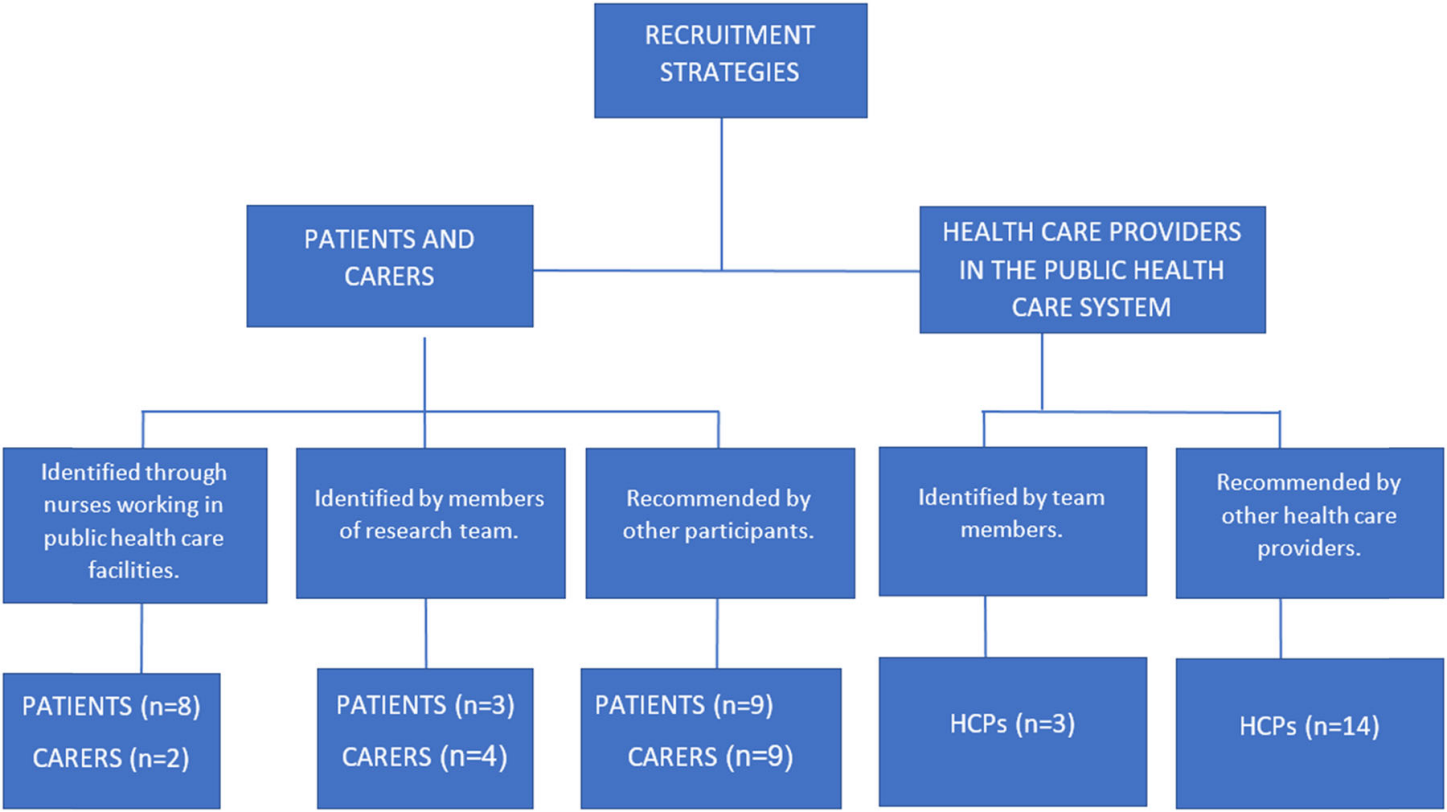
Diagrammatic representation of recruitment for the study. HCPs, healthcare providers.

Three recruitment strategies were used for identifying participants for the study (Figure 1). First, nurses informed eligible patients and carers attending the OP clinics of FHCs about the research study. Interested patients and carers who fulfilled the eligibility criteria were interviewed. Purposive sampling^36^ was used to identify eligible participants based on the following inclusion criteria: adult patients (18 years of age and older) and their carers with diabetes/hypertension or both, seeking care from public healthcare facilities or both public and private healthcare facilities. Second, patients, carers and HCPs were recruited through convenience sampling^37^ identified by members of the research team in Kerala. The convenience and purposive sampling were combined to ensure the participation of patients and carers from low socioeconomic groups (patients' and carers' self‐reported status of employment, education, housing and possession of a ration card (this is an identification card used by the public distribution system to identify families below the poverty line) using public and private healthcare facilities in the study. Third, purposive sampling was used to recruit HCPs working in public healthcare facilities in a range of districts, rural and urban locations and experience with electronic health records, and snowball sampling^38^ was used as a strategy to recruit more HCPs by asking HCPs at the end of interviews to recommend other HCPs.

### 2.4 Data collection

Semistructured interviews^34^ took place both face‐to‐face (*n* = 12) and by telephone (*n* = 40). The first author conducted all interviews in Malayalam using topic guides (Supporting Information: S1). These were piloted before data collection to ensure clarity. The patient and carer topic guide included open‐ended questions regarding healthcare visits to HCPs, diabetes and hypertension management at home, use of PHRs for information exchange, communication and self‐management. Patients and carers were interviewed separately. The HCP topic guide slightly differed to capture the context and organization of care for patients with diabetes and hypertension and existing systems for information exchange (PHR and electronic health records).

Face‐to‐face interviews with patients, carers and HCPs took place in an available quiet room at FHC. Telephone interviews were conducted at a convenient time for participants. All interviews were audio‐recorded and lasted between 15 and 60 min. The first author translated and transcribed the first five interviews from each group into English. A trained research assistant translated and transcribed the rest of the interviews into English. A local researcher (Postdoctoral Fellow in Sociology) who was familiar with the study settings and fluent in Malayalam and English checked for any translation and transcription errors in all the transcripts. Data collection continued until data saturation was reached within each of the groups.^39^ The interviews for each group (patients, carers and HCPs) were done sequentially. Data saturation is the point at which the researchers fully understand issues and when no further dimensions or insights into issues can be found.^39^ Achieving data saturation ensured that the findings were grounded in the experiences of key participant groups. First, patient interviews were done and data collection was stopped when no new information regarding PHRs was obtained. For example, the data on ‘how patients' carried their PHRs’ were explored until explanations for whether patients carry records or not, whether their behaviour differs in carrying PHRs for diabetes and hypertension and other conditions and the reasons why they carry them (or not) were obtained until no new information was added in the next interview. HCP interviews were done until no new information on recording and use of PHRs for communication, informational continuity and self‐management was obtained. For example, the documentation pattern of HCPs in the PHRs and the reasons for documenting or not documenting in PHRs were explored until no new data were obtained.

### 2.5 Data analysis

**Figure 2:**
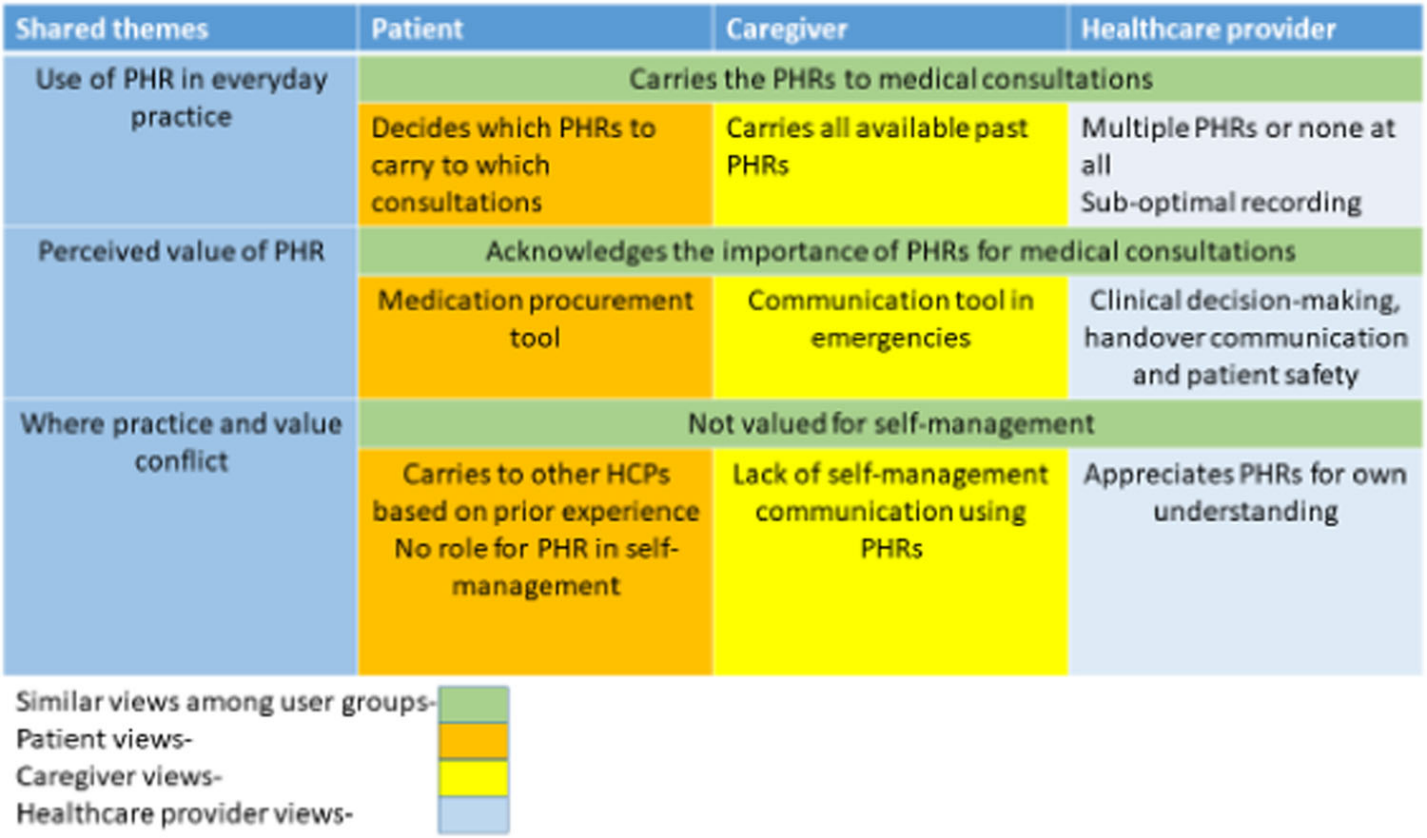
Conceptualizations of patient‐held records by participant groups. HCP, healthcare providers; PHR, patient‐held health record.

We used an iterative thematic approach^40^, ^41^, ^42^ to analysis, which focused on analysing interviews in their entirety and identifying themes related to patients', carers' and HCPs' experiences of using PHRs. A predominantly inductive approach to coding was followed. The first author coded three interviews from each group (patients, carers and HCPs) initially. The corresponding author then reviewed the transcripts and the generated codes. The first author coded the full data set manually. The patients', carers' and HCPs' data had been coded separately and codes were grouped into potential themes in Microsoft Excel. Potential themes were discussed and agreed upon with the team members. Not all the themes from the interview transcripts of patients, carers and HCPs are reported in this paper (will be published later). The themes and subthemes specifically about experiences with PHRs from patients, carers and HCPs were compared and contrasted and this focused analysis is reported in this paper. The results are organized under three headings (use of PHR in everyday practice, perceived value and where practice and value conflict) (Figure 2).

## 3 FINDINGS

The characteristics of patients, carers and HCPs interviewed (*n* = 52) are summarized in Table 2. The 20 patients were aged between 41 and 70 years and 8 of them had both diabetes and hypertension. Most of them were unemployed (*n* = 4) or engaged in minimum‐wage employment (*n* = 11). Carers were aged between 28 and 56 years of age; 10 of them had parents/in‐laws with diabetes/hypertension and others were spouses. HCPs included doctors (*n* = 13) and nurses (*n* = 4) in the public healthcare system. Their work experience ranged from less than a year to 20 years. Numerical pseudonyms are used when presenting quotes to ensure confidentiality.

**Table 2:** Study sample characteristics.

Patient identification	Age	Marital status	Literacy	Education	Occupation	Gender	Chronic NCD
IDI 1	70	married	Literate	8th standard (secondary)	Housewife	Female	Diabetes, hypertension
IDI 2	55	married	Literate	4th standard (lower primary)	Thozhilorup (a government‐sponsored minimum‐wage unskilled work for rural women)	Female	Diabetes, hypertension
IDI 3	64	married	Literate	5th standard (upper primary)	Manual labourer in the past	Male	Diabetes, hypertension and cardiovascular disease (CVD)
IDI 4	48	married	Literate	6th standard (upper primary)	Thozhilorup (a government‐sponsored minimum‐wage unskilled work for rural women)	Female	Diabetes, hypertension
IDI 5	42	married	Literate	4th standard (lower‐primary)	Skilled worker	Female	Diabetes, hypertension
IDI 6	51	married	Literate	10th standard (secondary)	Housewife	Female	Diabetes
IDI 7	52	married	Literate	3rd standard lower‐primary)	Housewife	Female	Diabetes, hypertension and CVD
IDI 8	68	Married	Literate	3rd standard lower‐primary)	Housewife	Female	Diabetes and hypertension
TP 1	54	Widow	Literate	Secondary education	Skilled worker	Female	Hypertension
TP 2	42	Married	Literate	Degree	Technical worker	Male	Diabetes mellitus
TP 3	53	Married	Literate	Secondary education	Skilled worker	Female	Diabetes mellitus
TP 4	52	Married	Literate	Secondary education	Skilled worker	Female	Diabetes mellitus
TP 5	64	Married	Literate	Completed secondary education	Skilled worker	Male	Diabetes mellitus
TP6	43	Married	Literate	Completed secondary education	Skilled worker	Female	Diabetes mellitus
TP 7	46	Married	Literate	Completed secondary education	Technical worker	Female	Hypertension
TP 8	57	Married	Literate	Degree	Administrative worker	Female	Hypertension
TP 9	62	Married	Literate	Diploma	Retired (Healthcare professional)	Female	Diabetes
TP 10	63	Married	Literate	Degree	Retired (Education/teacher)	Male	Diabetes and hypertension
TP 11	41	Married	Literate	Degree	Administrative worker	Male	Hypertension
TP 12	56	Married	Literate	Completed secondary education	Shop owner	Male	Diabetes
Carer 1	42	Married	Literate	Degree	Managerial worker	Male	Mother with diabetes
Carer 2	41	Married	Literate	PhD	Managerial worker	Female	Parents with hypertension and CVD
Carer 3	32	Married	Literate	Masters	Administrative worker (Research)	Female	Mother‐in‐law with diabetes, liver cirrhosis and cancer
Carer 4	31	Married	Literate	Masters	Healthcare professional (Research)	Female	Father with diabetes
Carer 5	32	Divorced	Literate	12th	Skilled worker	Female	Father and mother with diabetes CVD
Carer 6	48	Married	Literate	3rd standard	Skilled worker	Female	FIL and MIL with diabetes and hypertension
Carer 7	52	Married	Literate	8th standard	Unskilled worker	Female	Husband with diabetes
Carer 8	32	Married	Literate	Degree	Administrative worker	Female	Father
Carer 9	48	Married	Literate	12th (secondary school)	Housewife	Female	Husband with hypertension and CVD
Carer 10	56	married	Literate	10th standard	Unskilled worker	Female	Husband
Carer 11	45	Married	Literate	12th (secondary school)	Skilled worker	Female	Mother‐in‐law with diabetes
Carer 12	28	Single	Literate	10th standard	Skilled worker	Male	Father
Carer 13	32	Married	Literate	10th standard	Skilled worker	Male	Mother
Carer 14	56	married	Literate	12th (secondary school)	Skilled worker	Male	Husband
Carer 15	47	Married	Literate	12th (secondary school)	Housewife	Female	wife

Healthcare providers				
Identification	Gender	Qualification	Job designation	Years of experience
HCP1	Male	Graduate in Medicine	Doctor in PHC	1 year and 5 months
HCP2	Female	Graduate in Nursing	Staff nurse in PHC under NUHM	2 years
HCP3	Female	Diploma in Nursing	Staff nurse in PHC	3 years
HCP 4	Female	Postgraduate in Medicine	Doctor in CHC	12 years
HCP5	Female	Postgraduate in Medicine	Doctor in FHC	Less than a year
HCP 6	Female	Postgraduate in Medicine	Doctor in FHC	15 years
HCP 7	Female	Postgraduate in Medicine	Doctor in administrative cadre	6 years
HCP 8	Female	Diploma in Nursing	Staff nurse in FHC	4 years
HCP 9	Female	Diploma in Nursing	Staff nurse in PHC	4 years
HCP 10	Female	Graduate in Medicine	Assistant Surgeon	10 years
HCP 11	Male	Graduate in Medicine	Doctor in PHC	3 years
HCP 12	Male	Postgraduate in Medicine	Doctor in administrative cadre	less than a year
HCP 13	Female	Graduate in Medicine	Medical Officer in hospital	20 years
HCP 14	Male	Graduate in Medicine	Doctor in FHC	8 years
HCP 15	Male	Postgraduate in Medicine	Doctor in TH hospital	6 years
HCP 16	Female	Postgraduate in Medicine	Doctor in administrative cadre	3 years
HCP 17	Male	Graduate in Medicine	Doctor in FHC	2 years

Abbreviations: CHC, community health centre; FHC, family health centre; FIL, father‐in‐law; MIL, mother‐in‐Law; NUHM, National Urban Health Mission; PHC, primary health centre; TH, Taluk Hospital.

The first heading, ‘Use of PHR in everyday practice’, describes how each group engaged with the PHR in practice. The second heading, ‘Perceived value of PHR’, centres on how each user group's practices guided the value that they placed on PHRs. Specifically, this section demonstrates how previous healthcare visits influenced each user groups' perceptions. The third heading, ‘Where practice and value conflict’, explores how the practices and perceived values related to the PHR differ both within and between groups. Illustrative quotes are presented in Table 3.

**Table 3:** Themes and quotes.

Theme	Illustrative quotes
	We ask them to bring the notebook for next month's visit. We make sure that they carry them here (health centre) for monthly medication. Quote 1, HCP 14
Use of PHR in everyday practice	There is like a huge number (of people) in primary care, the dire need is to cater to them and finish the consultations soon. Then these issues as some doctors who work really hard will continue their work but some people are there don't work at all. Therefore, like some sort of hierarchy, which makes some work more, and they may have difficulty to give more care to patients. Then, there is no method anywhere, to improve the staffs those who work less. Even if they do not document well, there is no checks or penalty. In this situation, everywhere there is an issue like workload. It is only because of the difference in work not due to overwork. There are some systems where human resources are less, which is another issue. Quote 2, HCP 12, doctor with administrative and clinical responsibilities
	If I come for fever or something like that I will not bring this book [note‐book]. I will get the medicines written on the prescription paper (‘cheetu’). This book (note‐book) is only for things like sugar [local term for diabetes]. Everyone has to bring a book to get medicines [diabetes/hypertension medicines]. Quote 3 (IDI 6, Female, 42 years)
	But not everyone (patients) will carry records all time. There may be many papers also at times. Imagine having a long queue of patients outside your room and then someone brings in many papers, it will take time to go through them. I think for new patients we will have to sit through and check them, but with regular patients, it may be one or two here and there. Quote 4 HCP 1
	If somebody asks for some record for a check‐up, I have to search everywhere unless it is not arranged properly or kept in chronological order. If it is arranged in a file, my husband or mother‐in‐law can take it in my absence. Like for my mother‐in‐law, before we visited the Hepatologist, I arranged her records according to the date to understand the progress. Everything was available and it saved me a lot of going back and forth with records. However, I may not be always there in my home and I can tell them through the phone that I arranged it as on date. That much specific it is and so it helps me or others, each time with all the visits to doctors. Quote 5, Carer 3, 32 years, Female
Perceived value of PHR for themselves	But some patients may come without a prescription and tell three tablets for blood pressure, four tablets for some other problem, three yellow tablets, or round tablets. They are the more problematic persons for us. It becomes difficult then, they are having medicines for BP but we don't know which one and we may have to insist them to go and bring the papers. For them, it is their medicine, they probably don't realise that many tablets are round. Quote 6, HCP 6, doctor in FHC
	In situations, where a patient doesn't know the name of the drug or do not know if they have been taking medicines for BP, even if they say they are saying they take medicine and they don't know the name or dose or not have any documents like a past prescription, we consider him/her as a new patient. Quote 7, HCP 12, doctor in FHC
	Once a COPD (Chronic Obstructive Pulmonary Disease) patient came to FHC, we were not aware that he had this condition (COPD). The patient did not mention it nor did have any records with him. We (the doctor) prescribed him a particular antihypertensive. Later we understood he is a COPD patient. Then he came onto the next visit, with the prescription of a pulmonologist and we changed the anti‐hypertensive for him. So now, the whole treatment was affected. Quote 8, HCP 5
	At first, because the private hospital is nearby and I regularly visit the doctor there for my pressure (local term for hypertension). Since I came to diagnosed I had pressure I have been taking medicines from the same doctor, but it was expensive. Then I came here (FHC), mainly because this FHC has started giving medicines for pressure free. So I bring the book to show here and get the medicines. Quote 9, IDI6, Female, 51 years
	I was in the hospital for 30 days…. I used to go there (hospital) for my treatment sometimes, but it takes one whole day, travelling, and the queue and then somehow when we see the doctor, we just want them to write the medicines and go back. So then, I came here (FHC) and they gave me a book, wrote my details in them, and told me to bring it every time when I come here for medicines. Quote 10, IDI2, 55 years, Female
	It's better to have records with us, without records we won't have proof to tell or to show. In my case with our parents' means, they won't be remembering about their conditions [when visiting casualty]; they will be in a different emotion or situation when they reach hospital so they won't be able to communicate it properly. Quote 11, Carer 2, 40 years, Female
Where practice and value conflict	Yes, I do carry the paper. Mainly for the eye doctor to see how much is my ‘sugar’ [local term for blood glucose value and diabetes]. They usually ask for that. They will ask you what the previous value was or when was your last check‐up, things like that. Quote 12, IDI 6, Female, 42 years
	I thought that this is for them (healthcare providers) to write (‘ezuthi pathipikuga’). Quote 13, IDI 2, Female, 55 years
	Everyone knows there is very rush in the primary health centre (PHC). Initially, it will take more time, once the database (electronic health record) is fully activated, it will be very easy. Only at the pharmacy do they (patients/carers) get a printout, for the remaining [places] everywhere it is paperless. So patients need not carry any papers after eHealth is completed. Quote 14, HCP7, doctor in administration
	No, they don't ask like the names of medicines and for any papers for diabetes. They will just ask if I am taking any medicines regularly and then I tell them that I take medicines for pressure and cholesterol. Then they will say that is ok. Sometimes they will ask for medicine but I can't say that always. Mostly they don't ask. Quote 15, TP
	I ask the nurse when they check and write the BP in the book, like if it is normal. I don't look at the values but hearing from them if it is normal I am ok. Quote 16, IDI 5, Female, 51 years
	Yes, she (daughter‐in‐law) checks the test result also checks the medicines provided in the slip or the book. I mean she knows my medicines. Like if it is changed, increased, or decreased and she will tell me that it is decreased or not. Quote 17, IDI 5, Female, 51 years

Abbreviations: FHC, family health centre; HCP, healthcare providers.

### 3.1 Use of PHR in everyday practice

HCPs varied in their responses when describing how they used PHRs for documenting consultations and the extent of their information recording. Most HCPs described looking at PHRs if the patients bring them to consultations. HCPs requested and insisted on PHRs from patients with diabetes/hypertension, particularly for their monthly consultation. (Quote 1)

Some HCPs described spending time, especially when the patient (diabetic/hypertensive) is using their healthcare facility for the first time, collecting a detailed health history and documenting this in the PHR for future use. However, HCPs felt that their subsequent recording in PHRs was inadequate. HCPs reported finding suboptimal recording in PHRs when patients come for monthly consultations. Most HCPs cited a heavy patient load in OP settings as the reason for their own perceived inadequate documentation. However, one HCP cited other reasons for inadequate documentation by other HCPs such as the absence of monitoring of HCP documentation and a higher patient load on junior HCPs. (Quote 2)

Although HCPs described how patients did not always carry PHRs, all the patients participating in this study described carrying PHRs to FHC/public healthcare facilities when they visit health centres for monthly medication for diabetes or hypertension. Patients framed the process of carrying the books or records to the monthly consultations for diabetes or hypertension as their responsibility. However, it was also clear that they do not always carry all PHRs to other healthcare consultations. (Quote 3)

Against this background, HCPs discussed how having lots of different PHRs makes it difficult to locate the information needed or to take time to find it. (Quote 4)

In contrast to patients' decision‐making regarding which PHRs to carry, carers in the study described either carrying *all the past* PHRs themselves or encouraging the patient to do so for consultations. Carers felt that because the person they were caring for had multiple PHRs, they felt unqualified to choose which record to carry. Their views also included HCPs needing the medical information in the PHRs. One carer noted that she filed all her parents' PHRs chronologically and found that it was a great help in subsequent visits to HCPs. (Quote 5)

In everyday practice, thus, patients said that they brought diabetes/hypertension‐specific PHRs to diabetes/hypertension appointments, but did not carry them to other healthcare visits. In contrast, carers did not make such decisions and tended to carry all records to healthcare visits. HCPs requested PHRs from patients with diabetes/hypertension when they visit them for the first time and monthly diabetes/hypertension consultations. However, according to HCPs, recording in PHRs varies and is suboptimal. As such, each group of participants appeared to at least partially engage with PHRs in practice, albeit in different ways.

### 3.2 Perceived value of PHR for themselves

The perceived value of PHR by the users was based on their own PHR practices. These practices are attributed to the purpose that it serves for each group.

#### 3.2.1 Clinical decision‐making, handover communication and patient safety

HCPs regarded PHRs as an important tool for clinical decision‐making. HCPs described feeling more confident in managing those patients who had brought their PHR, due to having their medical history available in the PHR for review. HCPs thereby emphasized the value of having documented evidence of previous management and for preventing the creation of future gaps in information. HCPs felt that having documented information is particularly important when the patient is unable to communicate accurate details of their medicines. They also explained their lack of trust in the information communicated by patients as they felt that this might be affected by the recall. (Quote 6)

Three HCPs explained that when they encounter patients without a PHR or with missing information on past medications, they consider them as new patients. Thus, they begin the treatment with patients' current issues. (Quote 7)

HCPs reported that the value of PHRs lies in preventing medication errors and thus emphasized their importance in ensuring informational continuity and patient safety. (Quote 8)

Neither patients nor carers mentioned preventing medication errors or patient safety issues as motivating their carrying of PHRs to appointments.

#### 3.2.2 Medication procurement

For patients, the perceived value of their diabetes and/hypertension PHRs lay in medication procurement/medication refills. The value, therefore, was underpinned by the need to gain access to long‐term free medications. (Quote 9)

According to the patients, the staff at healthcare facilities advised them to carry the PHR when they visit the doctor to collect their medications (Quote 10). Some HCPs described how PHRs could act as a way of tracking medication procurement by patients. However, in contrast to the patients or carers, this was not the primary utility of PHRs described by the HCPs.

### 3.3 Emergency use

Carers play an important role in patients' care in Kerala, particularly in emergencies. They said that they would often replace ambulance‐based emergency medical systems and transport patients to hospitals in emergencies.

In contrast to patients and HCPs, carers framed the value of PHRs in terms of their use during emergencies. Not all carers in this study said that they accompanied patients to every HCP visit. Irrespective of this, all carers found it difficult to provide accurate descriptions of a patient's up‐to‐date medical information, including medications. Carers described carrying PHRs when they take patients to casualty or in cases when patients cannot explain their condition to HCPs. (Quote 11)

### 3.4 Where practice and value conflict

Under this heading, the participants' views on areas in which PHRs are being used in practice, but where they do not always identify the value of PHR for themselves, and also where the booklets' use differs from that which is intended are summarized.

Patients said that they carried PHRs to healthcare consultations even when they could not find value in this for themselves. Patients confirmed carrying them to healthcare visits when they had experienced HCPs requesting PHR. (Quote 12)

As described above, most patients considered the PHR important for medication procurement, and only very few patients described referring to PHRs as a reminder of their own daily medication intake. This is maybe one of the possible reasons why these PHRs are not communicated by HCPs as a self‐management tool to the patients and patients perceive PHRs as tools for the HCPs. (Quote 13)

HCPs reported that they did not use PHRs (even the ones with additional health information such as a menu plan) to direct patients and carers for follow‐up or self‐management at home. In addition, some HCPs referred to the implementation of electronic health records in the public health system, which would help patients, as patients do not need to carry any papers to healthcare visits. Some HCPs did not see the value of PHRs for patients/family members themselves, as they placed emphasis instead on information transfer across healthcare visits. (Quote 14)

Patients and HCPs had different views on medication information recall and therefore they differed in describing the value of PHRs for communicating about medicines. Some patients reported that most HCPs only asked if they had a condition such as diabetes or hypertension and if they are taking medication for them when going to healthcare visits other than for diabetes/hypertension so they did not think they would need to carry PHRs for DM/hypertension to other visits. (Quote 15)

Patients were asked whether they looked at their own lab results from the PHRs and most responded that they do not. Some patients reflected that their results were discussed with them by HCPs. For example, one patient described having a conversation with the nurses when they check blood pressure values in the PHR and whether the values were ‘normal’. Patients preferred having a conversation with HCPs rather than using their current PHRs for self‐monitoring. (Quote 16)

Some patients said that they find PHRs to be a source of medical information for carers. Most patients reported discussing the reports of diagnostic tests/lab tests with their carers. Similarly, most carers placed value on the medical information in PHRs, which enabled them to understand what happened at the doctor's appointment when they were not accompanying the patient. Even though patients appeared not to read or find value in using PHR for monitoring care, they did find some value in PHRs providing an opportunity for family members to discuss their health condition. (Quote 17)

## 4 DISCUSSION

This qualitative study explored similarities and differences in patient, carer and HCP perspectives on using PHRs for managing diabetes and hypertension in Kerala. These are vital actors in the management of patients with chronic diseases in India and most LMICs and, together, can ensure the provision of seamless, long‐term continuity of care to enable better health outcomes for the patients. Patients and carers reported carrying PHRs to consultations but patients made decisions on which PHRs to carry, based on the purpose of the healthcare visit. HCPs felt that their own documentation in records was inadequate due to the heavy patient load. Each of the user groups was seen to place a different value on the PHR, based on their own conceptualization of its importance for themselves. HCPs perceived PHRs as valuable for preventing medication errors and improving informational continuity and patient safety. Patients perceived PHRs to be valuable for them in procuring medicines for their conditions. Finally, carers perceived PHRs to be important for them in communicating patients' medical information to HCPs during emergencies. Therefore, patients and HCPs did not view current PHRs as a tool for self‐management. Carers felt that PHRs provide information for communication and self‐management at home. This is because the relationship between use in everyday practice and the value that each group places on PHR is complex.

Overall, our findings indicate that among our study participants, PHRs were being used mostly as information transfer tools across healthcare visits to the same provider for diabetes/hypertension consultations. However, owing to different values placed by patients and HCPs on PHRs and a lack of awareness of the use of PHRs by providers, the information carried by patients may not be comprehensive. Due to the divergent values of the different user groups, there are differences in use and lost opportunities for optimal use of PHRs that could lead to improved patient care and health outcomes.

### 4.1 Comparison with the existing literature

Previous studies of maternal PHRs from HIC reported improved communication between women and HCPs and improved involvement of women in their care.^43^ However, communication among the community and hospital HCPs has not improved using PHRs.^44^ Women from HICs and LMICs valued having their own medical information.^6^, ^43^ However, there is limited literature from LMICs on PHRs for diabetes and hypertension.^15^ Our study goes beyond previous research by exploring disconnects between HCP's, patients' and carers' current use and the value of PHRs and how they influence handover communication, information transfer and self‐management in Kerala. A mixed‐methods study carried out in Ireland to evaluate the use of PHRs in palliative care found that families used and valued PHRs more than patients or HCPs.^45^ While patients could find the value in using PHRs for communicating with families and HCPs, they did not use them in practice. The HCPs did not feel that PHRs helped much in facilitating decisions or communicating with patients, families and HCPs.^45^ Understanding disconnects in the use and value of PHRs could therefore inform modifications to PHRs and help develop support interventions that could enhance their use.

### 4.2 Patient safety

In our study, HCPs felt that the medical information in PHRs is valuable for clinical decision‐making, the prevention of medication errors and thus for enhancing patient safety. The providers' views of value, which are based on providing appropriate care, are consistent with previous literature.^46^ However, in our study, HCPs did not use PHRs for communicating with patients nor emphasized the importance of patients taking the PHRs to all HCPs, irrespective of the healthcare facility. Additionally, patients and carers with previous experience with HCPs requesting PHRs in the past tended to say that they take them to their subsequent consultations. However, not taking into account the potential for preventing medication errors or patient safety, patients carried records based on the purpose of their healthcare appointments. These findings are similar to a study carried out in the UK, which found that patients and carers were unaware of the purpose and value of carrying medication lists to consultations to enhance medication safety.^47^ This finding highlights the need for support interventions such as creating awareness regarding the purpose of PHRs to all stakeholders irrespective of health settings.

### 4.3 Informational continuity

Our study findings suggest that although patients viewed PHRs for diabetes/hypertension primarily for medication procurement, they took the PHRs to other provider consultations. A qualitative study from Australia reported differences in patient with long‐term conditions views on carrying PHRs to HCPs. Patients who actively participated in their health felt they would take PHRs to their providers. However, patients who were more passive in making decisions about health did not feel the need to carry their information in PHRs to their HCPs.^48^ Specifically, patients with previous experience with HCPs requesting PHRs in the past said they carried them to their subsequent consultations just that the HCP may ask for them again as they did in a previous visit. This shows that patients carrying records may not be an act of being empowered as suggested by previous literature,^48^, ^49^ rather the behaviour is a result of the external motivation provided by thinking that HCPs have asked them for the records in the past and hence may ask again. This finding does not imply that extrinsic motivation is of a lower utility to the use of PHR in practice by patients than intrinsic motivation. Locke and Schattke argue that external motivation can be viewed as a means to an end or doing something for future value.^50^ Similarly, in this study, patients carried PHRs to other consultations due to the external motivation provided by HCPs requesting PHRs, even when patients did not themselves consider their PHR necessary for communicating with HCPs. This finding points towards a reinforcing role for HCPs in improving patients and carers' carrying PHRs to healthcare visits and particularly important in health systems with minimal facility‐based records and lack of integrated electronic records.

Multiple PHRs led patients to make decisions on which PHR to take to the consultations, leading to suboptimal information transfer. Multiple PHRs posed difficulty in informational continuity for providers, as they may not have up‐to‐date information on all medications/diagnostic tests or other pertinent medical information that aid in preventing medication errors/duplication of tests. Therefore, our findings suggest an increasing risk for patient safety, especially for patients with multiple morbidities who are increasing in prevalence in Kerala^51^ and similar LMIC settings.^52^

### 4.4 Communication

Our findings show that the value carers placed on PHRs were different from that of patients. Most previous studies from HIC combine patients' and carers' views of the acceptability and usefulness of PHR together.^11^, ^47^ However, in this study, it was illustrated that there was a different primary value of PHR for carers. Carers valued PHRs for its importance in communicating patients' conditions and medications during an emergency referral to health centres when the carers need to act promptly and appropriately, often without the information known only to the patient themselves. Carers or family members in similar settings who are involved in patients' healthcare may find PHRs valuable.^53^

### 4.5 Patient engagement with PHRs

This study showed that most patients reported not reading or looking at the PHR themselves. One possible explanation the low levels of patients' engagement with their own PHRs is that patients view PHRs as documents for HCPs. Previous literature has shown that most patients in Kerala believe that HCPs act in the patient's best interest.^13^ Thus, there is an accepted notion that the documents written by HCPs need to be kept safe when instructed to do so since the doctor may ask for it next time. Additionally, since the patients are not given any instructions for how to use the PHRs for self‐management, and effort is not made to write notes in a way that is useful to patients so that generally they cannot read or appreciate the information in their own PHRs. This problem is particularly acute for patients with lower levels of education who may find it even more difficult to read and understand HCPs' notes or instructions in PHRs. The potential implications of these findings are significant for Kerala as well as LMICs, given that chronic disease patients have to be able to manage their own care if they are to avoid emergency crises, long‐term complications and ultimately to take the overall pressure on the healthcare services needed to manage these patients. There is evidence that if the patients are relying on verbal information alone, the comprehension and retention of information by patients is less than that written for them to take home, and without PHRs carers may also not be able to explain follow‐up or self‐management needs to patients or communicate with future HCPs.^54^, ^55^, ^56^ Additionally, for the patient's own use, previous studies from HIC have found that a lack of timely information regarding patient medical information such as treatment details, can increase the probability of adverse events.^57^, ^58^, ^59^

### 4.6 Implications for practice and research

Our findings indicate a need for the healthcare systems to consider the use of universal^14^ or standard PHR^60^ in continuity of care across multiple providers systems, for the prevention of medication errors and improving patient safety and for carers to support the patients during emergency acute crises. If PHRs are to contribute to handover, informational continuity and self‐management, policy makers and implementers need to recognize the potential divergences of use and value of PHRs to patients, carers and HCPs. Further, HCPs need training, implementation protocols and monitoring and supervision for better use of PHRs for improving continuity of care. Additionally, designing easy‐to‐use formats and creating awareness for the patients and carers to bring the PHRs during every visit to any provider may enable better information transfer across providers. Furthermore, empowering patients and carers to communicate with HCPs regarding recording notes in PHRs may ensure better handover communication.

Similar research on how different stakeholders use and value PHRs can help to reaffirm the areas in which PHRs may be developed. PHRs may be valuable for information transfer across multiple provider systems, for the prevention of medication errors, to improve patient safety and for carers to support patients during a health emergency. PHRs are potentially a low‐cost intervention that could have a significant impact on safer and more efficient healthcare for chronic NCD patients, irrespective of any electronic record system currently envisaged in India or elsewhere. Future research should go beyond the findings of this study to examine in more depth HCPs' perceptions about the role of PHRs in self‐management of patients with diabetes and hypertension.

### 4.7 Strengths and limitations

The use of qualitative methodology has revealed several key issues reflected in the user groups' use and perceived value of PHRs for handover communication. This is the first study to explore, compare and contrast the views of users of PHRs in Kerala. The study's credibility was strengthened using data triangulation,^61^ through interviews with patients, carers and HCPs. Our study findings present views of patients, and carers from lower socioeconomic strata visiting mostly public health facilities. Therefore, the results may be transferable to other low socioeconomic groups in other LMICs. The number of interviews conducted resulted in data saturation^62^ being achieved for each individual group.

Due to the varied nature of PHRs used by participants in the study, the findings cannot give insights into whether a treatment prescription is valued more than a comprehensive patient‐held notebook for its users. Further, the findings from the study do not show any difference for less literate patients' value for illustrated PHRs. Previous reports from maternal and child health have shown that graphically illustrated PHRs are valued more by less literate mothers. Moreover, previous studies from HIC show regardless of the type of PHR, proper use by HCPs and patients is more important for preventing medication errors.^47^ A reflexive approach was taken to consider the influence of L. J. who has had clinical training from Kerala. The multidisciplinary research team trained in anthropology, medical sociology and clinical backgrounds contributed to the analysis and interpretation of the findings to minimize bias.

## 5 CONCLUSION

We argue that any healthcare tool will perform differently in a complex healthcare system. Hence the success of maternal and child health records such as vaccination cards for information storage and transfer may not be achieved with a PHR for diabetes/hypertension in a pluralistic health system. Our exploration of the use and the perceived value placed on PHRs found that currently the PHR served each group differently who then placed a different value on PHRs. The utilitarian focus of the health system and HCPs in Kerala may lead to more disparity in health information transfer, and a more costly healthcare system as patient safety is compromised in the short‐run and complications and multimorbidities rise in the long‐term.

Quality of care improvement by better information transfer and patient safety should be a focus for managing people with NCDs in LMICs. Better healthcare governance for preventing fragmentation of care across private and public healthcare facilities and loss of information transfer needs to be prioritized. The current pattern of use of PHRs among participants in this study does not provide informational continuity across all HCPs. PHRs may be utilized as an adjunct to electronic health records. Further research is needed to understand better the type and content of PHR for people with long‐term conditions, and support interventions to ensure information exchange between HCPs in public and private health systems, and the efficient use of hard‐copy PHR along with electronic records^14^, ^63^ or development of an easily and universally accessible electronic PHR. A co‐design approach^64^, ^65^ involving meaningful consultation with patients, carers and HCPs for using PHRs could be a possible way to increase the engagement of patients.

## AUTHOR CONTRIBUTIONS

Linju Joseph, Sheila Greenfield, Semira Manaseki‐Holland, Panniyammakal Jeemon and Anna Lavis contributed to the study conception and design. Linju Joseph and Anna Lavis designed data collection tools. Semira Manaseki‐Holland, Sheila Greenfield, Anna Lavis and Panniyammakal Jeemon provided guidance in the overall design and delivery of the research. Linju Joseph collected the data. Linju Joseph, Sujakumari S. and Lekha T. R. prepared the data and data analysis was performed by Linju Joseph and Anna Lavis. The first draft of the manuscript was written by Linju Joseph and all authors contributed to critical revisions of the manuscript. All authors read and approved the final manuscript.

## CONFLICT OF INTEREST STATEMENT

The authors declare no conflict of interest.

## ETHICS STATEMENT

Ethical approval for the qualitative study was obtained from the Centre for Chronic Disease Control, New Delhi [CCDC_IEC_05_2019], and the University of Birmingham [ERN_18‐1933].

## Supporting information

Supporting information.

## Data Availability

Data are available on request from the authors. The anonymized interview transcripts that support the findings of this study are available from the corresponding author upon request.
