# Supplementary material for: Patients', carers' and healthcare providers' views of patient‐held health records in Kerala, India: A qualitative exploratory study
Source: Health Expect. 2023 Feb 13;26(3):1081–95. doi: 10.1111/hex.13721 (PMC10154823; doi:10.1111/hex.13721)
Supplement: Supplementary file 1 — Supporting information. [file HEX-26--s001.docx]

# S1 Interview guides

Patient topic guide

- Tell me why you have come to primary care today?

*Your symptoms, treatment and effects of your condition on daily life*

- Could you please tell me about how you came to know about your condition (“sugar/pressure”)?

*How did you seek care?*

- Could you tell me about your current treatment?

*Number of doctors involved, health care facilities, current medicines, the process of getting treatment, meeting doctors, taking blood pressure/blood sugar at facilities*

- What information regarding your condition have you received from health care providers (HCP)?

*About the condition, treatment, diet/physical activity advices verbal and documented*

- How do you feel about managing your condition?

*At home, involvement of family members, communication with HCPs, getting medicines, getting tests*

- Could you please tell me how you manage your health care visits?

*Do you go to other HCPs for sugar and pressure?*

- In your opinion, do you think HCPs need information from regarding your past medical conditions?

*Yes/No; then why?*

- Could you tell me how you manage the papers you get from your doctors?

*Storing, carrying across health care visits in the same facility, carrying to different HCPs and health care facilities, use at home, show to family members, do you look at what HCPs have documented?*

- How do you feel about using the papers given to you for managing your care?

*Does the papers from HCPs hold any value for you or not and why*

*Can you think of any factors which helps you to use the papers/booklet?*

- Could you tell me if you are able to use the information in the booklet such as previous blood pressure values or medication prescription to manage your condition in anyways?

*Communicating with doctors/nurses at primary centres or other health care facilities, look at the blood pressure/blood sugar values to see if they are improving or not, use it for managing medicines, communicating with family members*

Carer topic guide

- Could you please tell me about how your family member came to know about their condition (“sugar/pressure”)?
- Could you tell me about their current treatment?

*Number of doctors involved, health care facilities, current medicines, the process of getting treatment, meeting doctors, taking blood pressure/blood sugar at facilities*

- What information regarding their condition have you received from health care providers (HCP) when you have accompanied them?

*About the condition, treatment, diet/physical activity advices verbal and documented*

- Could you please tell me how you manage your family members’ health care visits?

*Do you accompany them? Do you ask details about the communication with HCPs? Do you encourage them go to other HCPs for sugar and pressure?*

- In your opinion, do you think HCPs need information from regarding your family members’ past medical conditions?

*Yes/No; then why? One or more examples when you needed the papers for HCPs*

- Could you tell me how you help your family member manage the papers from doctors?

*Storing, carrying across health care visits in the same facility, carrying to different HCPs and health care facilities, use at home, do you look at what HCPs have documented?*

- How do you feel about having the papers given to you/family member for managing care?

*Does the papers from HCPs hold any value for you or not and why*

*Can you think of any factors which helps you to use the papers/booklet?*

- Could you tell me if you are able to use the information in the booklet such as previous blood pressure values or medication prescription to manage your family members’ condition in anyways?

*Communicating with doctors/nurses at primary centres or other health care facilities, look at the blood pressure/blood sugar values to see if they are improving or not, use it for managing medicines, communicating with family members*

Healthcare provider topic guide

- Could you please walk me through your typical day like in FHC (or PHC/CHC/hospital)?
- Could you please tell me about how you manage patients with diabetes and hypertension in your centre?
- Could you please tell me if people mention access as an issue for their care?
- Could you tell me in your opinion issues relating to management of patients with diabetes and hypertension in your centre?
- Have you got a chance to look at patient’s lab reports?
- Could you please tell me what kind of notes patients bring to consultation?
- How do you currently manage any records brought by patients to FHC?

*Could you please tell me what do you normally do with other OP sheets/ reports brought by patients?*

- What written documentation do you provide the patients with diabetes and hypertension and other NCD patients?

*Any advice regarding how to use these documents for themselves, other HCPs*

- In your opinion, do patients take this book/papers to other providers?
- What do you think about not having documented patient’s health information from other providers?

*One or more examples when you needed the papers from other HCPs, how did you manage without past information?*

- In your experience and opinion can patients explain what has happened in the previous doctor visit?
- In your experience how do you provide information on caring for themselves to patients/carers?

*Verbal information, booklet/recorded information*

- Could you please describe your experience with recording in electronic health records?

*What was the best thing about it? What was the most difficult? How does it affect information retrieval and handover communication? What are your thoughts on electronic health records current implementation?*
